# Epithelium-Specific Ets-Like Transcription Factor 1, ESE-1, Regulates ICAM-1 Expression in Cultured Lung Epithelial Cell Lines

**DOI:** 10.1155/2015/547928

**Published:** 2015-06-21

**Authors:** Zhiqi Yu, Jun Xu, Jinbao Liu, Jing Wu, Chan Mi Lee, Li Yu, Jim Hu

**Affiliations:** ^1^State Key Lab of Respiratory Disease and Guangzhou Institute of Respiratory Disease, The First Affiliated Hospital of Guangzhou Medical University, Guangzhou Medical University, Guangzhou 510120, China; ^2^Protein Modification and Degradation Laboratory, Department of Pathophysiology, Guangzhou Medical University, Guangdong, China; ^3^Physiology & Experimental Medicine Program, Hospital for Sick Children, Toronto, ON, Canada M5G 1X8; ^4^Department of Laboratory Medicine and Pathobiology, University of Toronto, Toronto, ON, Canada M5S 1A8; ^5^Department of Pediatrics, Guangzhou First People's Hospital, Affiliated to Guangzhou Medical University, Guangzhou, Guangdong 510180, China

## Abstract

Cystic fibrosis (CF) patients suffer from chronic airway inflammation with excessive neutrophil infiltration. Migration of neutrophils to the lung requires chemokine and cytokine signaling as well as cell adhesion molecules, such as intercellular adhesion molecule-1 (*ICAM-1*), which plays an important role in mediating adhesive interactions between effector and target cells in the immune system. In this study, we investigated the relationship between *ICAM-1* and epithelium-specific ETS-like transcription factor 1 (*ESE-1*) and found that *ICAM-1* expression is upregulated in cell lines of CF (IB3-1) as well as non-CF (BEAS-2B and A549) epithelial origin in response to inflammatory cytokine stimulation. Since *ESE-1* is highly expressed in A549 cells without stimulation, we examined the effect of *ESE-1* knockdown on *ICAM-1* expression in these cells. We found that *ICAM-1* expression was downregulated when *ESE-1* was knocked down in A549 cells. We also tested the effect of *ESE-1* knockdown on cell-cell interactions and demonstrate that the knocking down *ESE-1* in A549 cells reduce their interactions with HL-60 cells (human promyelocytic leukemia cell line). These results suggest that *ESE-1* may play a role in regulating airway inflammation by regulating *ICAM-1* expression.

## 1. Introduction

Airway inflammation is a hallmark of the cystic fibrosis (CF) lung disease. The airways of CF patients are initially colonized by viruses, fungi, or bacteria, including* Staphylococcus aureus*,* Haemophilus influenzae,* and* Klebsiella pneumonia* [1]. Most patients later become infected with mucoid strains of* Pseudomonas aeruginosa* and some with* Burkholderia cepacia* [2].

In CF patients, the number of neutrophils and the levels of cytokines such as tumor necrosis factor-*α* (TNF-*α*), interleukin- (IL-) 6, and IL-8 in the airways are increased compared to non-CF individuals [3, 4]. Cultured CF lung epithelial cells (IB3-1) show downregulation of the anti-inflammatory cytokine IL-10 and an exaggerated upregulation of IL-8 in response to a variety of external stimuli, such as TNF-*α* and bacterial products [5, 6]. Overproduction of IL-8 is likely a major cause of excessive neutrophil infiltration, since IL-8 is a potent chemoattractant for neutrophils [7].

Neutrophil migration in response to inflammatory stimuli requires cell adhesion molecules, such as intercellular adhesion molecule-1 (*ICAM-1*, also known as CD54) [8, 9]. Migration of neutrophils out of the vascular system occurs in distinct phases: rolling, firm adhesion, and transmigration [10]. Four types of cell adhesion molecules are involved in this process, namely, E-selectin,* ICAM-1*, vascular cell adhesion molecule-1 (VCAM-1), and platelet-endothelial cell adhesion molecule-1 (PECAM-1). Neutrophil rolling is the first step of the migration process and E-selectin is the key molecule involved in slowing down the circulating neutrophils. This step is critical to ensure firm adhesion of neutrophils to the endothelial cell layer. Firm adhesion is mediated through* ICAM-1* expressed on endothelial cells, which interacts with CD11a/CD18 (LFA1) or CD11b/CD18 (Mac-1) as counter-receptors on neutrophils [11]. The final phase of transmigration of neutrophils through the endothelium is triggered by PECAM-1 and VCAM-1 [10]. Currently, the mechanism by which neutrophils migrate to the airway lumen is unclear, but they are thought to travel through the intercellular space [12, 13]. Other cell adhesion molecules such as ICAM-2 and ICAM-3 are also involved in the migration of monocytes [14] or dendritic cells [15].

*ICAM-1* is a 114 kD inducible surface glycoprotein that belongs to the immunoglobulin superfamily [9] and it plays an important role in innate and adaptive immune responses [16]. Although the role of* ICAM-1* in endothelial cells as well as in adaptive immunity [17–20] is well established, the function of epithelial* ICAM-1* during inflammation is not fully understood. Since epithelial* ICAM-1* is expressed on the airway lumen [21–24], a role for leukocyte transmigration is not expected. On the other hand, cell adhesion studies [25, 26] indicate that epithelial* ICAM-1* is important for leukocyte homing. Because neutrophils and macrophages are enriched at the sites of injury or inflammation, it is possible that homing of these cells is part of the resolution of inflammation.

Among the adhesion molecules,* ICAM-1* may play a more important role in the infiltration of leukocytes during airway inflammation. For example, Hubeau et al. performed quantitative analysis of inflammatory cells infiltrating the CF airway mucosa in lung tissues collected at the time of transplantation and found that* ICAM-1*, but not VCAM-1 or E-selectin, was overexpressed on the epithelium surface [27]. In addition, a recent* in vitro* study also showed that* ICAM-1* is expressed in a higher percentage of cultured airway epithelial cell lines (IB3-1, C38 and BEAS-2B) than other cell adhesion molecules, such as VCAM-1 or E-selectin [28].

*ICAM-1* is expressed at a very low level in airway epithelial cells. Interestingly, CF-deficient airway epithelial cells have a slightly higher basal level of* ICAM-1* expression [28]. Upon stimulation with proinflammatory cytokine (e.g., TNF-*α* or IL-1*β*) [29, 30] or other stimulatory substances (e.g., LPS) [31],* ICAM-1* expression is significantly induced in both human primary bronchial epithelial cultures and epithelial cell lines. This induction is mediated by activation of nuclear factor-kappa B (NF-*κ*B) signaling transduction pathway. In addition,* ICAM-1* induction can also be mediated through the STAT signaling pathway since IFN-gamma can significantly elevate its expression in epithelial cells [16]. CF airways have chronic inflammation, which contributes to the overexpression of* ICAM-1* [27]. Since epithelial* ICAM-1* may be critical for neutrophil homing and epithelial killing, it is important to understand its regulation and function in airway epithelial cells in order to identify potential drug targets for the CF lung disease.

The E26 transformation-specific (ETS) family of transcription factors is characterized by a highly conserved 85 amino acid DNA binding domain, which is known as the ETS domain [32]. It is comprised of 27 and 26 members in humans and mice, respectively. The ETS domain is usually located in the carboxyl-terminal region of the protein as a winged helix-turn-helix structural motif and binds to purine-rich DNA that has a core consensus sequence of GGAA/T- within the promoter and enhancer regions of target genes [33]. ETS transcriptional factors act as both positive and negative regulators of gene expression in various biological processes, such as cellular proliferation, differentiation, apoptosis, metastasis, hematopoiesis, and angiogenesis [34]. Although many of ETS family members are expressed in nonepithelial cells, such as hematopoietic and endothelial cells,* ESE-1* is mainly expressed in epithelial-rich tissues, such as lung, kidney, stomach, small intestine, colon, pancreas, trachea, salivary gland, prostate gland, mammary gland, uterus, and skin [35], but it can be upregulated in nonepithelial cells by proinflammatory cytokines such as TNF-*α* and IL-1*β*. Our previous work has shown that* ESE-1* can be highly induced in epithelial cells by inflammatory cytokines [36].

In this study, we investigated the regulation of* ICAM-1* expression by* ESE-1*. Here we demonstrate that the expression of both* ICAM-1* and* ESE-1* is upregulated in human bronchial epithelial cells (BEAS2B), CF cells (IB3-1), and lung cancer cells (A549) by inflammatory cytokines. We also show that* ICAM-1* expression is downregulated upon* ESE-1* knockdown in A549 cells and that* ESE-1* regulates the* ICAM-1* expression at the transcriptional level. Finally, we demonstrate that the downregulation of* ICAM-1* by knocking down* ESE-1* in A549 cells results in a reduced capacity of A549 cells to interact with HL-60 cells.

## 2. Materials and Methods

### 2.1. Cell Culture and Reagents

Human bronchial epithelial cells (BEAS-2B), human lung carcinoma cells (A549), and CF bronchi epithelial cells (IB3-1) were cultured in Dulbecco's Modified Eagle's Medium (DMEM) medium (Invitrogen) supplemented with 10% fetal bovine serum (FBS). Recombinant human tumor necrosis factor-*α* (TNF-*α*) and interleukin-1*β* (IL-1*β*) were obtained from R&D Systems (Minneapolis, MN) and reconstituted in phosphate buffered saline (PBS) containing 0.1% bovine serum albumin (BSA). For cytokines stimulation, TNF-*α* and IL-1*β* were used at 10 ng/mL each.

### 2.2. Viral Transduction and shRNA-Mediated Gene Knocking Down

*ESE-1* gene was knocked down in A549 cells using a shRNA helper-dependent adenoviral vector expressing two shRNAs from murine U6 gene promoter as described previously [36]. A C4HSU empty vector was used as control. For viral transduction, A549 cells were seeded at 2 × 10^5^ cells per well in 6-well plates overnight, and then cells were transduced at 40–60% confluency with viral vector at 2500 particles/cell (or 50 moi) under serum-free conditions for two hours, followed by addition of media to a final concentration of 10% FBS. We usually achieved near 100% transduction at this vector concentration. Cells were then collected for protein extraction or RNA isolation at desired time points.

### 2.3. Transient Transfection and Luciferase Reporter Assay

For cell transfection and cotransfection experiments, BEAS-2B cells were seeded in 6-well plates and transfected at 50–60% confluency by using PolyFect transfection reagent (Qiagen) according to the manufacturer's protocol. After 24 h, cells were harvested and luciferase activity was measured using a Dual Lucifcerase kit (Promega) and a luminometer (EG&G Berthold, BadWildbad, Germany) as described previously [37].

### 2.4. RNA Analysis

Cells were lysated and RNAs were extracted by using RNA spin Mini kit (GE Healthcare) according to manufacturer's instructions. Total RNA (1 *μ*g) was reverse transcribed using random hexamers and SuperScript II reverse transcriptase (Invitrogen), and then the resulting template (20 ng cDNA) was used for each real-time PCR reaction (ABI Prism 7700, Applied Biosystems). Primers for human* ESE-1* and* ICAM-1* were purchased from ACGT, Toronto. For relative quantification, PCR signals were compared between groups after normalization with GAPDH as an internal reference. Fold changes were calculated according to Livak and Schmittgen [38]. Primers used were hICAM1-F: GGAAGGTGTATGAACTGAGCAA, hICAM1-R: GGAGTCCAGTACACGGTGA, hese1-F: GGCGTCTTCAAGTTCCTGCG, hese1-R: CTCCCGTTTGTAGTAGTACCT, hGAPDH-F: GAAGGTGAAGGTCGGAGTC, hGAPDH-R: GAAGATGGTGATGGGATTTC.

### 2.5. Western Blotting Analysis

Cell lysates were run on an 8% SDS-polyacrylamide gel at 10 *μ*g each lane and transferred to nitrocellulose membrane (Bio-Rad) following electrophoresis. The membrane was then blocked with Tris-buffered saline with Tween-20 (TBST) containing 5% milk and probed with goat anti-human* ICAM-1* antibody (R&D System, Minneapolis, USA) at 1 : 1000 dilution and rabbit anti-human* ESE-1* antibody (R&D System, Minneapolis, USA) at 1 : 5000 dilution. Rabbit anti-human GAPDH antibodies (Trevigen, Gaithersburg, USA) were used at 1 : 3000 as a protein loading control. The horseradish peroxidase-conjugated secondary antibodies for rabbit anti-goat IgG were from Kirkegaard & Perry Laboratories and for goat anti-rabbit IgG were from Bio-Rad Laboratories. Detection of proteins was performed with enhanced chemiluminescence reagents (Amersham Pharmacia Biotech, Baie-d'Urfe, CA).

### 2.6. Cell Adhesion Assay

At day 0, 1 × 10^6^ A549 cells were seeded on a 10 cm dish and virus vector particles were added at 100 moi (5000 particles per cell) for 1 hour in serum-free media. Fetal bovine serum was then added to final concentration of 10%. At day 5, the treated cells were harvested and seeded at 250,000 to each well of a-24-well plate. The reason for waiting for 5 days was to minimize the influence of inflammatory cytokines induced by the viral transduction. For visualization of cell-cell interactions, HL-60 cells were labeled with carboxyfluorescein succinimide ester (CFSE) at 5 *μ*M in the cell culture for 5 min at 37°C and then washed 3 times with RPMI 1640. The labeled HL-60 cells were put back in the cell culture condition for 24 hours. The A549 cells were then stimulated with IL-1*β* and TNF-*α* each at 10 ng/mL for 6 hours and labeled HL-60 cells were activated with 12-*O*-tetradecanoylphorbol-13-acetate (TPA) at 5 ng/mL for 4 hours. After washing both cells, 1 million HL-60 cells in 0.5 mL of RPMI 1640 were added to each well of the treated A549 cells under cell culture conditions. Following adhesion for 1 hour, nonattached HL-60 cells were washed away with 1x PBS for 3 times at room temperature. Cells were fixed with 1% Paraformaldehyde in 1x PBS for 10 min and washed 3 times with 1x PBS. Cells were photographed using a Leica Fluorescence Microscope (model, DMIRE2).

### 2.7. Statistical Analysis

Data were analyzed by using Mann-Whitney* U* test (Kruskal-Wallis test for more than two groups). *P* < 0.05 was considered statistically significant.

## 3. Results

### 3.1. *ESE-1* and* ICAM-1* Expression Are Upregulated in BEAS2B, IB3-1, and A549 Cells in Response to Inflammatory Cytokines

**Figure 1:**
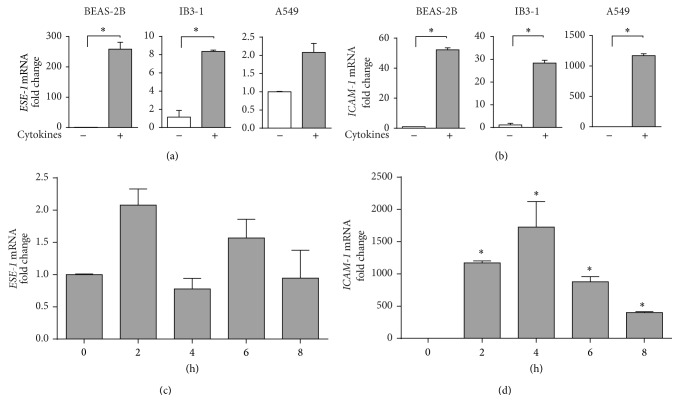
Induction of* ESE-1* and* ICAM-1* mRNA expression by TNF-*α* and IL-1*β* in different cell lines. (a, b)* ESE-1* and* ICAM-1* mRNA expression in BEAS2B, IB3-1, and A549 cells after stimulation with TNF-*α* and IL-1*β* (10 ng/mL each) for 2 hours. The mRNA expression levels were determined by real-time quantitative RT-PCR and the fold of change was based on the mRNA level of non-induced cells. Data were normalized to GAPDH and values were shown in 2^−ΔΔCT^ as the mean ± SD, *n* = 3, ^*^ *P* < 0.05. Statistics was performed as described in Materials and Methods. (c, d)* ESE-1* and* ICAM-1* mRNA expression in A549 cells at different time points following cytokine stimulation. Cells were lysed at time points as indicated after TNF-*α* and IL-1*β* (10 ng/mL each) stimulation. The fold of change was based on the expression level at time 0 hour. Data were collected and analyzed as described in (a) and (b).

To examine whether both* ESE-1* and* ICAM-1* are upregulated under proinflammatory conditions, BEAS-2B, IB3-1, and A549 cells were stimulated with a combination of proinflammatory cytokines TNF-*α* and IL-1*β* (10 ng/mL each) for 2 hours, and their mRNA levels were assessed by qRT-PCR. As shown in Figures 1(a) and 1(b), levels of mRNA for both* ESE-1* and* ICAM-1* were increased significantly (except for* ESE-1* expression in A549 cells). We also observed a higher level of* ESE-1* mRNA expression in A549 cells than BEAS-2B and IB3-1 cells. To further investigate at which time point* ESE-1* and* ICAM-1* start responding to cytokine stimulation, we completed a time course study. As shown in Figures 1(c) and 1(d), the* ESE-1* mRNA expression peaked at 2 hours after stimulation in A549 cells while* ICAM-1* mRNA expression reached a plateau at 4 hours after stimulation.

### 3.2. *ICAM-1* Expression Is Downregulated by the* ESE-1* Knockdown in A549 Cells

**Figure 2:**
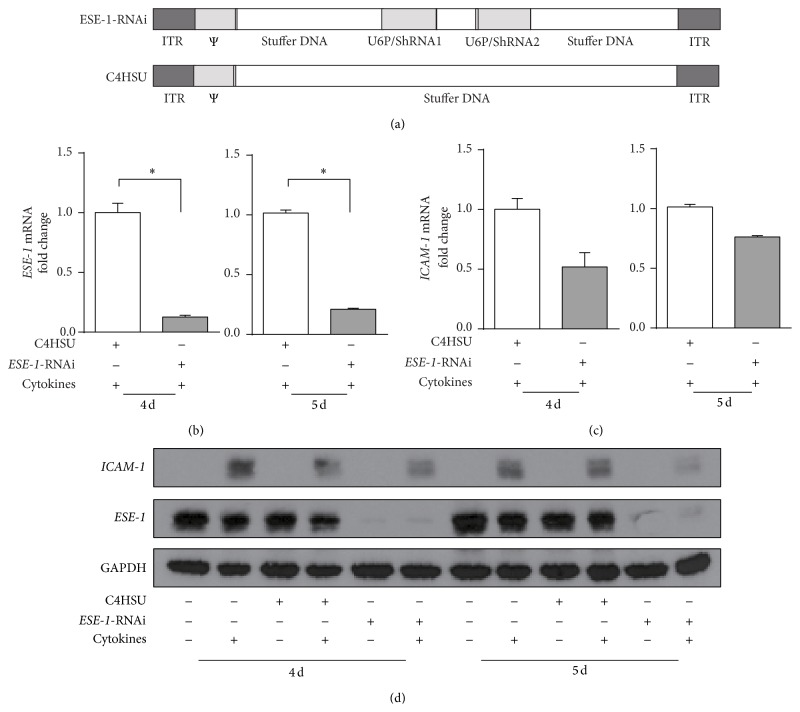
Effects of* ESE-1* knockdown in A549 cells on the expression of* ICAM-1*. (a) Schematic diagrams of the helper-dependent adenovirus vectors (HD-Ad) that were used for the* ESE-1* knockdown experiments. ESE-1-RNAi expresses two shRNAs from the murine U6 gene promoter, and C4HSU is used as an empty vector control which does not express any transgene. ITR: inverted terminal repeat; Ψ: packing signal. (b, c)* ESE-1* and* ICAM-1* mRNA expression in A549 cells 4 and 5 days after transduction with ESE-1-RNAi vector compared to that of C4HSU. Both groups were stimulated with TNF-*α* and IL-1*β* at 10 ng/mL each for 2 hours before cell lysis. The mRNA expression levels were normalized to GAPDH and the values were presented in 2^−ΔΔCT^ as the mean ± SD, *n* = 3, ^*^ *P* < 0.05. Statistics was performed as described in Materials and Methods. (d) A representative western blot analysis of* ICAM-1* expression with (+) and without (−) ESE-1-RNAi, compared to that of the C4HSU vector control group on day 4 and day 5 after transduction. Since* ICAM-1* levels were very low, subgroup of cells were stimulated with TNF-*α* and IL-1*β* (10 ng/mL each) for 16 hours to visualize ICMA-1 protein expression before cell lysis.

Since both* ESE-1* and* ICAM-1* mRNA expression were upregulated when stimulated by proinflammatory cytokines and given that* ESE-1* is a transcription factor, we questioned whether* ICAM-1* expression is transcriptionally regulated by* ESE-1*. We used a helper-dependent adenovirus (HD-Ad) vector to knockdown* ESE-1* gene expression in A549 cells and examined the changes in* ICAM-1* expression. We selected A549 cells for the knockdown experiment, as these cells constitutively express relatively high levels of* ESE-1*, at both mRNA and protein levels. As expected, we found a significant reduction of* ESE-1* mRNA following HD-Ad vector transduction (Figure 2(b)). Interestingly,* ICAM-1* expression was also downregulated compared to the empty vector (C4HSU) control group (Figure 2(c)). We examined* ESE-1* and* ICAM-1* expression at days 4 and 5 after transduction because viral vectors alone can induce the production of proinflammatory cytokines that can last for more than two days after viral transduction. We also used longer time points for cytokine stimulation in protein analysis (16 h) than in mRNA analysis (2 h), as changes in protein expression lag that of the mRNA.

### 3.3. *ESE-1* Gene Regulates* ICAM-1* Expression at the Transcriptional Level

**Figure 3:**
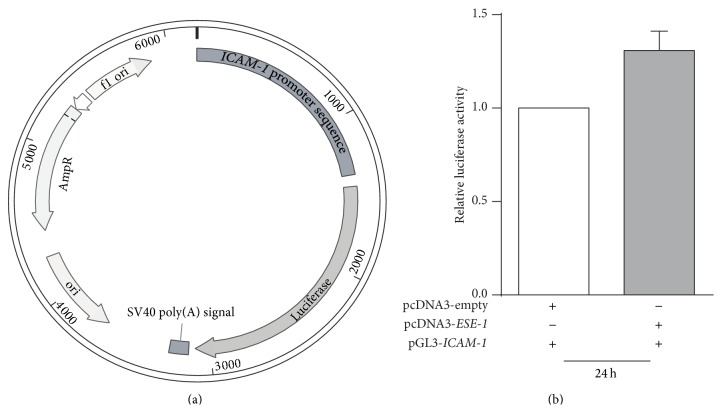
Luciferase assay on BEAS2B cells after cotransfection with pcDNA3-ESE-1 and PGL3-ICAM-1 vector. (a) Schematic diagram of pGL3-ICAM-1 promoter reporter plasmid that contains an* ICAM-1* promoter sequence expressing the luciferase gene in the pGL3-Basic vector (Promega). (b) Luciferase activity assay. BEAS-2B cells were cotransfected with pGL3-ICAM-1 luciferase reporter plasmid and pcDNA3-ESE-1 or pcDNA3-empty vector [37]. The luciferase activity was measured 24 h after transfection.

To further investigate the regulatory mechanism, we developed an* ICAM-1* promoter reporter assay to examine the role of* ESE-1* in the regulation of* ICAM-1* expression. As shown in Figure 3(a), we made the reporter construct by inserting a 1.4 kb human* ICAM-1* promoter in front of the luciferase reporter gene in pGL-3-basic vector (Promega). BEAS-2B cells were cotransfected with the* ICAM-1* promoter luciferase reporter plasmid and pcDNA3-ESE-1 or pcDNA3-empty as an empty vector control. BEAS-2B cells are noncancerous human bronchial epithelial cells which express minimal basal levels of* ESE-1*, allowing us to specifically overexpress the protein by transfection. Twenty-four hours after cotransfection, the luciferase activity was measured and as shown in Figure 3(b), the level of luciferase activity was increased in the* ESE-1* overexpression group compared to empty vector control group, suggesting that* ESE-1* regulates* ICAM-1* expression at the transcriptional level.

### 3.4. Knockdown of* ESE-1* Expression in A549 Cells Reduces Binding of HL-60 Cells

**Figure 4:**
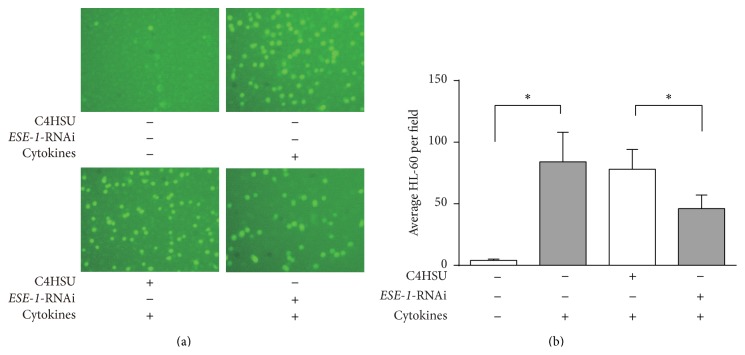
Cell adhesion assay of the effect of* ESE-1* knocking down on HL-60 binding to A549 cells. (a) Images showing HL-60 cells attached to A549 cells at 40x magnification. (b) Quantification of cell binding. The average number of HL-60 cells per field was compared among groups with* ESE-1* knocking down or C4HSU empty vector transfection or nontransfected groups with or without cytokine stimulation (TNF-*α* and IL-1*β* at 10 ng/mL each). Cell numbers were counted under a microscope and data from six wells were presented as mean ± SD, ^*^ *P* < 0.05.

Since* ICAM-1* is involved in cell-cell interactions, we performed a cell adhesion assay to investigate whether knockdown of* ESE-1* gene could affect cellular adhesion. We found that, following transduction with ESE-1-RNAi viral vector, A549 cells had less binding of HL-60 cells compared with C4HSU empty vector transduced group (Figure 4).

## 4. Discussion

In response to environmental perturbations, airway epithelia produce and release a variety of inflammatory cytokines, including TNF-*α*, IL-1, IL-6, and IL-8 [39]. A number of studies have shown that the epithelium of patients who have airway inflammatory diseases is structurally and functionally altered, and that bronchial epithelial cells that are isolated from patients with asthma or cystic fibrosis (CF) express increased levels of cytokines (IL-8, IL-25) [40, 41]. It is thus important to fully understand the gene regulation in airway epithelium in order to alleviate airway inflammatory diseases.

In this study, we show that both* ESE-1* and* ICAM-1* mRNAs are upregulated in lung epithelial cell lines after treatment with TNF-*α* and IL-1*β*. However, while both genes can be activated by the NF-*κ*B [42], we questioned whether there was any regulatory relationship between the two. The regulation of* ICAM-1* gene expression is primarily at the transcriptional level by several signaling pathways including protein kinase C (PKC), mitogen activated protein (MAP) kinase (JNK, ERK, and P38), and NF-*κ*B. The human* ICAM-1* gene promoter contains binding sites for many transcription factors, including AP-1, C/EBP, Ets, NF-*κ*B, STAT, and Sp1 [42]. Since* ESE-1* is a member of Ets family transcription factors which shares DNA binding sites, we decided to examine whether* ESE-1* regulated* ICAM-1* expression. To investigate the relationship between these two genes, we used helper-dependent adenovirus vector containing shRNA-ESE-1 to knockdown* ESE-1*. We observed that* ICAM-1* expression in A549 cells was indeed decreased after knocking down* ESE-1*.

To further investigate on the mechanism of this relationship between* ESE-1* and* ICAM-1*, we used* ICAM-1* promoter luciferase reporter assays to determine whether this upregulation was at the transcription level. Upon cotransfecting the luciferase reporter plasmid with an* ESE-1* gene expressing plasmid into BEAS-2B cells, we observed an increase in luciferase activity when* ESE-1* gene was overexpressed compared to the cells cotransfected with an empty vector plasmid. This result suggests that* ESE-1* regulates* ICAM-1* expression at the transcriptional level. However, we noted that the level of upregulation was not dramatic, but under chronic inflammatory conditions, a small change in* ICAM-1* levels may have a significant impact on disease progression.

We also performed a cell adhesion assay to investigate whether knocking down* ESE-1* could affect cell adhesion of epithelial cells. We show that, following transduction with ESE-1-RNAi vector, A549 cells exhibited less binding of HL-60 cells compared to groups of transduced with C4HSU empty vector or untransduced. This suggests that knocking down* ESE-1* in A549 cells causes downregulation of* ICAM-1* gene expression which in turn results in a reduced binding interaction between HL-60 and A549 cells.

*ICAM-1* is a key molecule that contributes to the control of inflammatory process. Although our finding of* ESE-1* regulating* ICAM-1* expression in this work is interesting, there are limitations to its clinical applications. First,* ESE-1* knocking down was carried out only in A549 cells, not in primary CF lung epithelial cells. Second, similar experiments have not been performed in any CF animal model. Future studies on this regulation in other model systems will be important for the development of effective anti-inflammatory strategies suitable for therapeutic intervention of inflammatory conditions such as CF. Since* ESE-1* is an transcription factor, it could be a potential drug target for screening small molecules that inhibit its expression.

## References

[1] Pilewski J. M., Frizzell R. A. (1999). Role of CFTR in airway disease. *Physiological Reviews*.

[2] Govan J. R. W., Deretic V. (1996). Microbial pathogenesis in cystic fibrosis: mucoid *Pseudomonas aeruginosa* and *Burkholderia cepacia*. *Microbiological Reviews*.

[3] Khan T. Z., Wagener J. S., Bost T., Martinez J., Accurso F. J., Riches D. W. H. (1995). Early pulmonary inflammation in infants with cystic fibrosis. *The American Journal of Respiratory and Critical Care Medicine*.

[4] Bonfield T. L., Panuska J. R., Konstan M. W. (1995). Inflammatory cytokines in cystic fibrosis lungs. *American Journal of Respiratory and Critical Care Medicine*.

[5] Li J., Johnson X. D., Iazvovskaia S., Tan A., Lin A., Hershenson M. B. (2003). Signaling intermediates required for NF-kappaB activation and IL-8 expression in CF bronchial epithelial cells. *The American Journal of Physiology—Lung Cellular and Molecular Physiology*.

[6] Venkatakrishnan A., Stecenko A. A., King G. (2000). Exaggerated activation of nuclear factor-*κ*B and altered I*κ*B-*β* processing in cystic fibrosis bronchial epithelial cells. *The American Journal of Respiratory Cell and Molecular Biology*.

[7] Strieter R. M. (2002). Interleukin-8: a very important chemokine of the human airway epithelium. *The American Journal of Physiology—Lung Cellular and Molecular Physiology*.

[8] Diamond M. S., Staunton D. E., de Fougerolles A. R. (1990). ICAM-1 (CD54): a counter-receptor for Mac-1 (CD11b/CD18). *Journal of Cell Biology*.

[9] Diamond M. S., Staunton D. E., Marlin S. D., Springer T. A. (1991). Binding of the integrin Mac-1 (CD11b/CD18) to the third immunoglobulin-like domain of ICAM-1 (CD54) and its regulation by glycosylation. *Cell*.

[10] Beck-Schimmer B., Schimmer R. C., Pasch T. (2004). The airway compartment: chambers of secrets. *News in Physiological Sciences*.

[11] Butcher E. C. (1991). Leukocyte-endothelial cell recognition: three (or more) steps to specificity and diversity. *Cell*.

[12] Kidney J. C., Proud D. (2000). Neutrophil transmigration across human airway epithelial monolayers: mechanisms and dependence on electrical resistance. *The American Journal of Respiratory Cell and Molecular Biology*.

[13] Liu L., Mul F. P. J., Kuijpers T. W., Lutter R., Roos D., Knol E. F. (1996). Neutrophil transmigration across monolayers of endothelial cells and airway epithelial cells is regulated by different mechanisms. *Annals of the New York Academy of Sciences*.

[14] Carreno M.-P., Chomont N., Kazatchkine M. D. (2002). Binding of LFA-1 (CD11a) to intercellular adhesion molecule 3 (ICAM-3; CD50) and ICAM-2 (CD102) triggers transmigration of human immunodeficiency virus type 1-infected monocytes through mucosal epithelial cells. *Journal of Virology*.

[15] van Kooyk Y., Geijtenbeek T. B. H. (2002). A novel adhesion pathway that regulates dendritic cell trafficking and T cell interactions. *Immunological Reviews*.

[16] Chang Y.-J., Holtzman M. J., Chen C.-C. (2002). Interferon-*γ*-induced epithelial ICAM-1 expression and monocyte adhesion. Involvement of protein kinase c-dependent c-Src tyrosine kinase activation pathway. *The Journal of Biological Chemistry*.

[17] Gaglia J. L., Greenfield E. A., Mattoo A., Sharpe A. H., Freeman G. J., Kuchroo V. K. (2000). Intercellular adhesion molecule 1 is critical for activation of CD28-deficient T cells. *Journal of Immunology*.

[18] Shaw S. K., Ma S., Kim M. B. (2004). Coordinated redistribution of leukocyte LFA-1 and endothelial cell ICAM-1 accompany neutrophil transmigration. *The Journal of Experimental Medicine*.

[19] Somersalo K., Anikeeva N., Sims T. N. (2004). Cytotoxic T lymphocytes form an antigen-independent ring junction. *Journal of Clinical Investigation*.

[20] Thompson P. W., Randi A. M., Ridley A. J. (2002). Intercellular adhesion molecule (ICAM)-1, but not ICAM-2, activates RhoA and stimulates c-fos and rhoA transcription in endothelial cells. *Journal of Immunology*.

[21] Burns A. R., Takei F., Doerschuk C. M. (1994). Quantitation of ICAM-1 expression in mouse lung during pneumonia. *Journal of Immunology*.

[22] Guzman J., Izumi T., Nagai S., Costabelt U. (1994). ICAM-1 and integrin expression on isolated human alveolar type II pneumocytes. *European Respiratory Journal*.

[23] Kang B. H., Crapo J. D., Wegner C. D., Letts L. G., Chang L. Y. (1993). Intercellular adhesion molecule-1 expression on the alveolar epithelium and its modification by hyperoxia. *The American Journal of Respiratory Cell and Molecular Biology*.

[24] Madjdpour C., Oertli B., Ziegler U., Bonvini J. M., Pasch T., Beck-Schimmer B. (2000). Lipopolysaccharide induces functional ICAM-1 expression in rat alveolar epithelial cells in vitro. *The American Journal of Physiology—Lung Cellular and Molecular Physiology*.

[25] Beck-Schimmer B., Madjdjpour C., Kneller S. (2002). Role of alveolar epithelial ICAM-1 in lipopolysaccharide-induced lung inflammation. *European Respiratory Journal*.

[26] Frick A. G., Joseph T. D., Pang L., Rabe A. M., St. Geme J. W., Look D. C. (2000). Haemophilus influenzae stimulates ICAM-1 expression on respiratory epithelial cells. *Journal of Immunology*.

[27] Hubeau C., Lorenzato M., Couetil J. P. (2001). Quantitative analysis of inflammatory cells infiltrating the cystic fibrosis airway mucosa. *Clinical & Experimental Immunology*.

[28] Tabary O., Corvol H., Boncoeur E. (2006). Adherence of airway neutrophils and inflammatory response are increased in CF airway epithelial cell-neutrophil interactions. *The American Journal of Physiology—Lung Cellular and Molecular Physiology*.

[29] Bloemen P. G. M., van den Tweel M. C., Henricks P. A. J. (1993). Expression and modulation of adhesion molecules on human bronchial epithelial cells. *The American Journal of Respiratory Cell and Molecular Biology*.

[30] Krunkosky T. M., Fischer B. M., Martin L. D., Jones N., Akley N. J., Adler K. B. (2000). Effects of TNF-*α* on expression of ICAM-1 in human airway epithelial cells in vitro: signaling pathways controlling surface and gene expression. *The American Journal of Respiratory Cell and Molecular Biology*.

[31] Fakler C. R., Wu B., McMicken H. W., Geske R. S., Welty S. E. (2000). Molecular mechanisms of lipopolysaccharide induced ICAM-1 expression in A549 cells. *Inflammation Research*.

[32] Oikawa T., Yamada T. (2003). Molecular biology of the Ets family of transcription factors. *Gene*.

[33] Lelièvre E., Lionneton F., Soncin F., Vandenbunder B. (2001). The Ets family contains transcriptional activators and repressors involved in angiogenesis. *International Journal of Biochemistry and Cell Biology*.

[34] Seth A., Watson D. K. (2005). ETS transcription factors and their emerging roles in human cancer. *European Journal of Cancer*.

[35] Oettgen P., Alani R. M., Barcinski M. A. (1997). Isolation and characterization of a novel epithelium-specific transcription factor, ESE-1, a member of the ets family. *Molecular and Cellular Biology*.

[36] Wu J., Duan R., Cao H. (2008). Regulation of epithelium-specific Ets-like factors ESE-1 and ESE-3 in airway epithelial cells: Potential roles in airway inflammation. *Cell Research*.

[37] Newnham C. M., Hall-Pogar T., Liang S. (2010). Alternative polyadenylation of MeCP2: influence of cis-acting elements and trans-acting factors. *RNA Biology*.

[38] Livak K. J., Schmittgen T. D. (2001). Analysis of relative gene expression data using real-time quantitative PCR and the 2^−∆∆*C*_*T*_^ method. *Methods*.

[39] Takizawa H. (1998). Airway epithelial cells as regulators of airway inflammation (Review). *International Journal of Molecular Medicine*.

[40] Aldallal N., McNaughton E. E., Manzel L. J. (2002). Inflammatory response in airway epithelial cells isolated from patients with cystic fibrosis. *The American Journal of Respiratory and Critical Care Medicine*.

[41] Beale J., Jayaraman A., Jackson D. J. (2014). Rhinovirus-induced IL-25 in asthma exacerbation drives type 2 immunity and allergic pulmonary inflammation. *Science Translational Medicine*.

[42] van de Stolpe A., van der Saag P. T. (1996). Intercellular adhesion molecule-1. *Journal of Molecular Medicine*.

